# Medication abortion during the COVID-19 pandemic in France: A research based on the French national health insurance database

**DOI:** 10.1371/journal.pone.0295336

**Published:** 2024-02-07

**Authors:** Justine Chaput, Valentine Becquet, Pierre-Louis Bithorel, Elodie Baril, Elise de La Rochebrochard, Magali Mazuy

**Affiliations:** 1 Institut national d’études démographiques (INED), Aubervilliers, France; 2 CRIDUP, Université Paris 1 Panthéon-Sorbonne, Aubervilliers, France; 3 Ecole des hautes études en démographie (HED), Paris, France; 4 CESP U1018, Institut national de la santé et de la recherche médicale (INSERM), Villejuif, France; UCSD: University of California San Diego, UNITED STATES

## Abstract

**Objectives:**

During the COVID-19 pandemic in France, abortion was recognized as an essential service that cannot be delayed, and such care was therefore presumed to be maintained. The aim is to analyze the changes in the practice of abortion in 2020 to identify the consequences of the two lockdowns and the effects of the extension of the legal time limit.

**Methods:**

We analyzed the data collected by the French national health insurance system, which covers 99% of the population. All women who had an elective abortion, either surgical or with medication, in France in 2019 and 2020 were included in the study. Trend changes in abortions were analyzed by comparing the ratio of the weekly number of abortions in 2020 with the weekly number in 2019.

**Results:**

Both 2020 lockdowns were followed by a drop in abortions, particularly after the first and stricter lockdown. This may be explained not by an abrupt shutdown of access to abortion services, but rather by a decrease in conceptions during the lockdown weeks. The decrease was more marked for surgical abortions than for medication abortions in a hospital setting, and less so for medication abortions in non-hospital settings. Moreover, the proportion of the latter type of abortions continued to increase, showing the reinforcement of a previous trend.

**Conclusions:**

Our findings indicate that expanding the legal time limit for abortion, diversifying the settings where abortions can be performed and the range of abortion providers help to facilitate access to this fundamental reproductive care.

## Introduction

To contain the spread of COVID-19, many countries have promulgated lockdowns and/or curfews [1]. For example, in 2020, France experienced a first very strict two-month lockdown (March 17–May 11, 2020), followed by a less strict six-week lockdown and curfew (October 30–December 15, 2020). A distinction was made between “essential” and “non-essential” healthcare: the latter could legally be postponed or cancelled contrary to the former. However, access to all healthcare services, including essential care such as reproductive care that, by definition, cannot be delayed, was restricted in practice by curfews and lockdowns. Pre-existing barriers to abortion were reinforced, such as the need to perform the procedure within the legal time limit or difficulty in reaching an abortion service, in particular for women living in remote areas. In addition, being confined at home may have obstructed some women’s need to keep the pregnancy secret [2].

While human rights and fundamental liberties were strongly restricted by the COVID-19 emergency measures [3], some governments (such as Hungary, Poland and several US states) used the sanitary crisis to designate abortion as nonessential care, thus restricting access even further. Others (such as Canada, Finland, Portugal and the UK) implemented policies to facilitate access to abortion [4–8]. In France, elective abortion can be performed up to 14 weeks of gestation. The time limit depends on the method: 14 weeks for surgical abortion (which can only be practiced in hospital settings), 9 weeks for medication abortions performed in hospital settings and 7 weeks for medication abortions in non-hospital settings. In April 2020, among other temporary emergency measures, new regulations were promulgated to extend access to medication abortions practiced in non-hospital settings up to 9 weeks, to improve healthcare provision.

The aim of this paper was to analyze the trend change in abortions in 2020 compared with the previous year, in both hospital and non-hospital settings and for both medication and surgical methods, and to identify the consequences of the lockdowns. In order to observe the impact of the legislative change, medication abortion was analyzed in detail, as well as the proportion of abortions carried out during the two-week extension of the legal time limit.

## Material and methods

### Data source

The French national health insurance database covers 99% of the resident population. It exhaustively records individual reimbursements of all drugs, medical devices, laboratory tests, medical procedures, private and public hospital stays, and healthcare. It also provides information on patients: sex, age, commune of residence, and registration with health insurance for low-income patients. This data source has been described in detail elsewhere [9]. Access to French health insurance databases is controlled by strict national legislation and is granted only by a personal time-limited authorization for a specific research project.

### Study population

Since rules of reimbursement are elaborated in a national system and abortion care is fully covered, the study population included all women who had an abortion in France between January 1, 2019, and December 31, 2020, that was registered in the French national health insurance database (S2 Table). Telemedicine abortions concerned probably only 700 women in 2020 and we consider that the codes used to identify them are not efficient (based on the cost, it might include other types of care). Therefore, we chose to not include them in our analysis.

### Prespecified outcome

Abortion was defined as an elective abortion including surgical abortion (legally practiced only in hospital settings) and medication abortion (legally practiced both in hospital and non-hospital settings). Spontaneous miscarriages and stillbirths were not included.

#### Sample size

The sample size was 460,015 abortions: 234,065 in 2019 and 225,950 in 2020.

### Statistical analysis

The weekly number of abortions was estimated for each week of the years 2020 and 2019 (the reference year). Changes in abortions were analyzed by comparing the ratio of the weekly number of abortions in 2020 with the weekly number in 2019 (number of abortions in 2020 for 100 abortions in the same week in 2019).

All statistical analyses were performed using SAS Enterprise Guide software (version 7.15; SAS Institute Inc., Cary, NC, USA).

### Patient and public involvement statement

Patients were not directly involved in the development, design and conduct of this study: the analysis was exclusively based on the French national health insurance database. Data are pseudonymized and all results are produced at an aggregated level.

## Results

During the study period, the number of elective abortions declined by 3% from 234,000 in 2019 to 226,000 in 2020. However, the proportion of abortions varied by setting and technique. While the number of abortions performed in hospital decreased by 17% for surgical abortions and by 2% for medication abortions, those performed in non-hospital settings (medication abortions only) rose by 9% (S1 Table).

**Fig 1:**
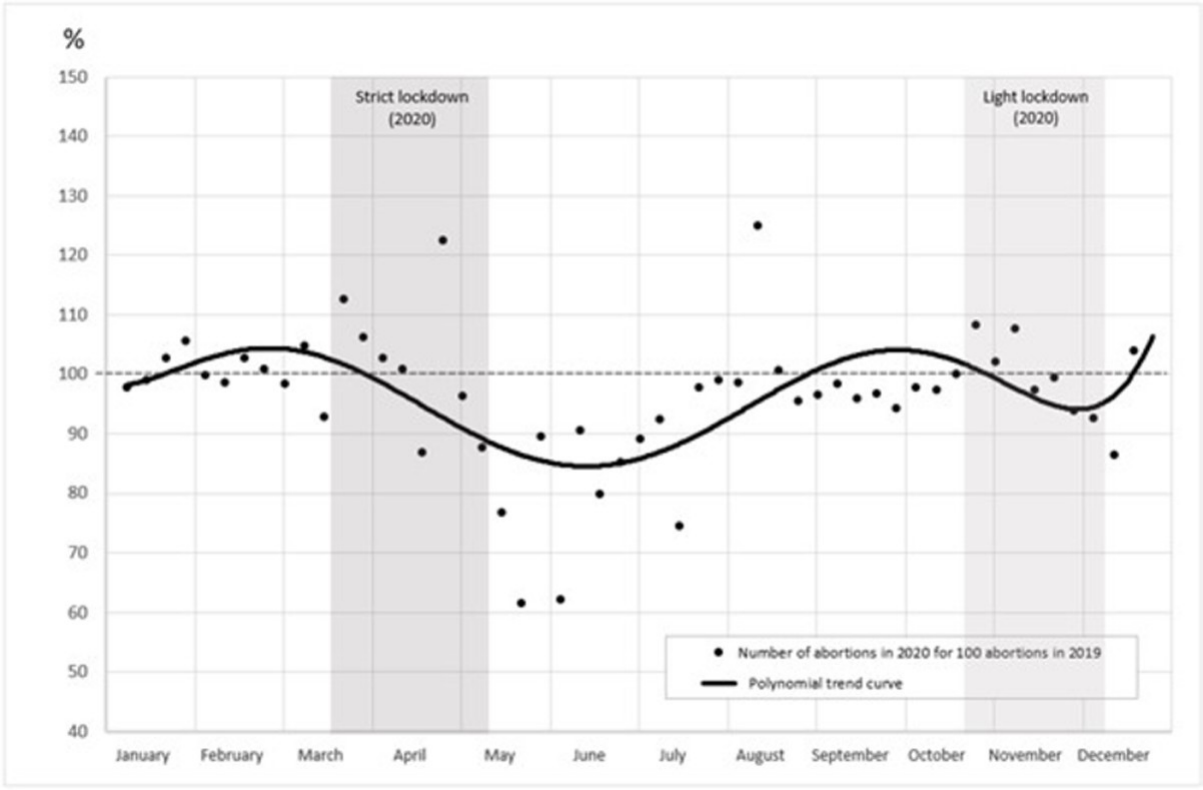
Ratio of total weekly abortions in 2020 compared with 2019 (%). *Notes*: Coverage: abortions in 2019 and 2020 in the whole of France. Source: Authors’ calculations based on the French health insurance database (PMSI-MCO & CNAM).

**Fig 2:**
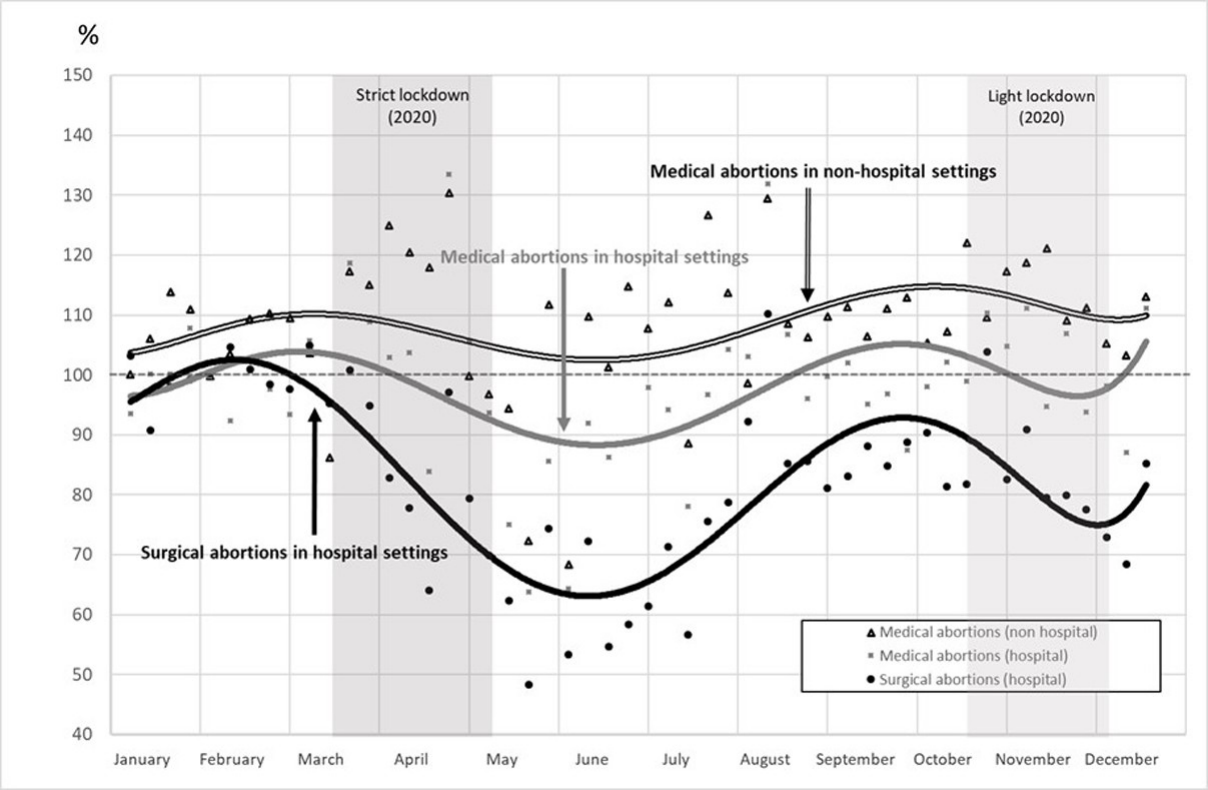
Ratio of weekly abortions in 2020 compared with 2019, by method and setting (%). *Notes*: Coverage: abortions in 2019 and 2020 in the whole of France. Source: Authors’ calculations based on the French health insurance database (PMSI-MCO & CNAM).

Before the first lockdown, in January and February, the weekly abortion ratio varied between 98 and 108, with a mean of 102 indicating a trend to a slight increase of 2% in 2020 compared with 2019 (Fig 1). Two weeks after the beginning of the first lockdown, the ratio decreased to below 100 and dropped by 38% in June compared with 2019 (the two lockdown periods are shown in gray on the graph). At the end of August 2020, the number tended to return to the 2019 level, until a second decrease that occurred during the second lockdown. These changes were observed for all abortion methods and locations (hospital and non-hospital settings), although to different degrees (Fig 2). Concerning all abortions performed in hospital settings, the ratio decreased from March to June and then increased until October. It again declined during the second lockdown before increasing in December. However, the ratio of surgical abortions decreased sharply and stayed below 100 between March and the end of 2020, whereas the trend of medication abortions was smoother, with slight variations around 100 during the same period. In contrast, the ratio of medication abortions in non-hospital settings always remained above 100 with variations related to the sanitary crisis, although they were less marked than for other types of abortions.

**Fig 3:**
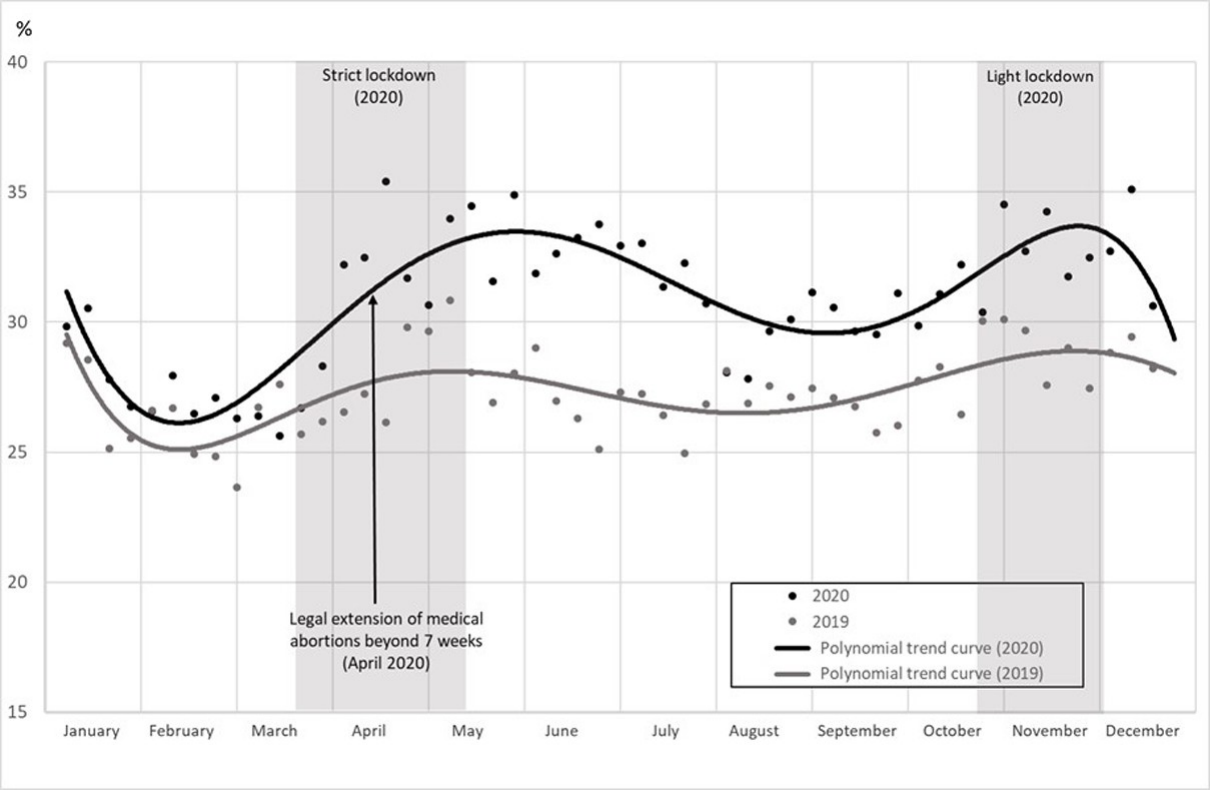
Proportion of medication abortions in non-hospital settings among total weekly abortions (%). *Notes*: Coverage: abortions in 2019 and 2020 in the whole of France. The extension of the legal time limit was implemented in 2020, therefore no abortion could be practiced under this condition in 2019. Source: Authors’ calculations based on the French health insurance database (PMSI-MCO & CNAM).

The proportion of medication abortions performed in non-hospital settings, among all abortions performed in 2020 (black line) and in 2019 (gray line), is displayed in Fig 3. From January to the beginning of March, the 2020 line was slightly above the 2019 line, showing a small increase in the proportion of abortions performed in non-hospital settings. The gap between the two lines then increased, particularly after the legal extension of the time limit for medication abortions in non-hospital settings. This extension from 7 to 9 weeks was implemented on April 14, 2020. The gap between the 2020 and 2019 lines slowly narrowed after the first lockdown but increased again with the second lockdown.

**Fig 4:**
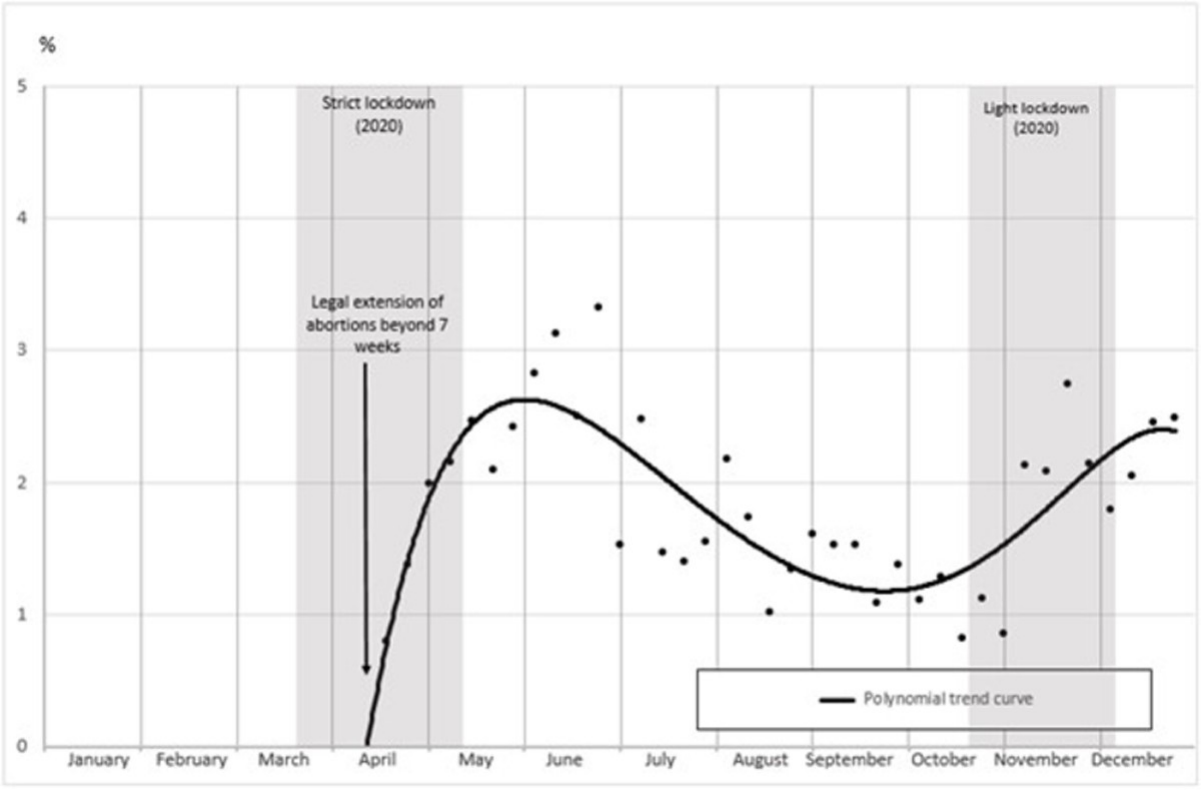
Proportion of abortions performed between 7 and 9 weeks in non-hospital settings in 2020 (%). *Notes*: Coverage: abortions between 7 and 9 weeks of gestation among medication abortions in non-hospital settings in 2020 in the whole of France. Source: Authors’ calculations based on the French health insurance database (PMSI-MCO & CNAM).

The proportion of medication abortions performed in non-hospital settings within the two-week legal extension is presented in Fig 4. These 8–9 week abortions increased up to around 3% after the first lockdown, decreased during the summer and increased again during the second lockdown. It means that right after the first lockdown, up to 1 in 7 abortions practiced between 7 and 9 weeks was provided in non-hospital settings, thanks to the new legislation.

These results reflect the effect of the lockdowns–in particular the first one–and the effect of the extension of the legal time limit. They also highlight different dynamics between abortions depending on the method and setting.

## Discussion

The annual number of abortions declined in 2020, with a decrease concentrated within two periods. Both 2020 lockdowns were followed by a significant drop in abortions. The first, strict lockdown coincided with a progressive decrease of abortions that lasted even after the end of lockdown. This time lag suggests that this lockdown did not lead to an abrupt shutdown of access to abortion care providers, thus we may consider other hypotheses than difficulties in accessing abortion services. This drop might be explained by a decrease in conceptions during the weeks of the lockdown [10–12]. A similar drop in births was in fact observed in January and February 2021, 9 months after the first lockdown (March 17–May 11, 2020), but not in the following months [13].

More specifically, the proportion of medication abortions in non-hospital settings increased while it decreased for those performed in hospitals. The drop was even more noticeable for surgical abortions. The proportion of medication abortions in non-hospital settings was thus higher in 2020 than in 2019, and this increase was observed during the whole year. There may have been a shift from hospital to non-hospital settings, probably because throughout the year hospitals were overburdened with COVID-19 care. However, it followed the long-term trend toward medication methods that is mainly explained by the fact that several professionals can perform medication abortions: general practitioners and gynecologists, and more particularly midwives, who are authorized to perform abortions in non-hospital settings since 2016 [14]. This general trend was reinforced during the pandemic, partially because of the extension of the legal time limit from 7 to 9 weeks. The partial takeover by the non-hospital care network was effective because this network had been developed previously.

The proportion of medication abortions performed after 7 weeks reached its peak during the weeks directly following the lockdowns. It may be related to the drop of abortions in hospitals: this drop could be partially explained by a decrease of the proportion of abortions after 7 weeks–these abortions having been perhaps partially performed in non-hospital settings as the law allowed it then. These findings suggest that the adaptation of the law may have had a direct impact on healthcare supply, particularly in a crisis context. Other countries also adapted their legislation to facilitate access to abortions and observed similar results. For example, in the United Kingdom, the diffusion of telemedicine had a significant impact on both abortion access and the possibility of performing abortions earlier in pregnancy [15, 16]. In France, telemedicine was very rare and was therefore not developed in our analysis. However, healthcare providers and women seeking abortion are beginning to show more interest in this type of care [17, 18].

### Strengths and limitations

Our analysis was based on the French health system databases, which are considered to be exhaustive. However, a small number of cases may be missing, particularly for telemedicine as its registry system is a complex one. Telemedicine requires further in-depth study, as it has shown interesting results for women internationally [16, 19, 20]. We were unable to analyze barriers in access, as women who did not succeed in accessing abortion care were not included. These women may have had to find another solution if the legal time limit had been exceeded. Our findings would benefit from complementary work on the most vulnerable social groups. Detailed analysis by geographical region would also be relevant because medication abortion care in non-hospital settings is not uniformly available throughout the country [10].

### Social implications

As the risk of health complications rises with the number of weeks of gestation [21], abortion care cannot be delayed. Emergency measures can take time to be implemented in regions with fewer abortion providers and facilities. They require the development and setting up of new regulations, with information made available to women and healthcare providers, who also may need to learn a new medical protocol. Relying only on such emergency measures may therefore not be sufficient to ensure access to care. Indeed, the legal time limit for medication abortion in non-hospital settings was extended after legislation was proposed. Other proposed permanent legislation, such as the two-week extension for elective abortion (from 12 to 14 weeks), was eventually promulgated in March 2022.

## Conclusions

Our results showed on the one hand that both 2020 lockdowns were followed by a drop in abortions, which might be explained by a decrease in conceptions during the lockdown weeks. On the other hand, the proportion of medication abortions in non-hospital settings has been increasing in the long term. This trend was further reinforced in 2020, particularly during the lockdowns, and the extension of the legal time limit from 7 to 9 weeks has contributed to this dynamic. This extension was much anticipated both because hospital practitioners were overburdened and because the lockdowns certainly led to increased mean gestational age at the time of abortion. Expanding the legal time limit for abortion, extending the right to perform an abortion to midwives, diversifying the settings where abortions can be performed, and more generally, reducing the number of intermediaries and steps in the procedure [22]. these measures all help to facilitate access to this fundamental reproductive care. Furthermore, if a wide and established network for abortion care had not already existed, these measures could not have been set up so quickly, as healthcare providers needed to adapt to changes in practice. Our results point out the importance of legislation during a crisis such as the COVID-19 pandemic and of its continuation in a non-crisis context.

## Supporting information

S1 Table Annual abortions by setting and technique in 2019 and 2020. *Notes*: Coverage: abortions in 2019 and 2020 in the whole of France. Source: Authors’ calculations based on the French health insurance database (PMSI-MCO & CNAM). In 2020, the total number of abortions may include a small number of acts related to one single woman. The unit of analysis is based on all care procedures that were covered, rather than on individuals.
(DOCX)

S2 Table List of codes for abortions. * Homogeneous groups of patients (G*roupe homogène de malades*, *GHM*) are a French administrative classification used to measure hospitals’ activity. They are the similar to the Diagnosis related groups (DRG) used in the United States. ** The *Classification commune des actes médicaux* (*CCAM*) is the French classification of medical procedures.
(DOCX)

## References

[1] Perra N. Non-pharmaceutical interventions during the COVID-19 pandemic: A review. Phys Rep. 2021;913:1–52. doi: 10.1016/j.physrep.2021.02.001 33612922 PMC7881715

[2] De Zordo S, Mishtal J, Zanini G, Gerdts C. Consequences of gestational age limits for people needing abortion care during the COVID-19 pandemic. Sexual and Reproductive Health Matters. 2020;28(1):1818377. doi: 10.1080/26410397.2020.1818377 33003990 PMC7887947

[3] Han E, Tan MMJ, Turk E, et al. Lessons learnt from easing COVID-19 restrictions: an analysis of countries and regions in Asia Pacific and Europe. The Lancet. 2020;396(10261):1525–1534. doi: 10.1016/S0140-6736(20)32007-9 32979936 PMC7515628

[4] Bateson DJ, Lohr PA, Norman WV, et al. The impact of COVID-19 on contraception and abortion care policy and practice: experiences from selected countries. BMJ Sex Reprod Health. 2020;46(4):241–243. doi: 10.1136/bmjsrh-2020-200709 32788180

[5] Moreau C, Shankar M, Glasier A, Cameron S, Gemzell-Danielsson K. Abortion regulation in Europe in the era of COVID-19: a spectrum of policy responses. BMJ Sex Reprod Health. 2021;47(4):e14–e14. doi: 10.1136/bmjsrh-2020-200724 33093040 PMC8515109

[6] Bayefsky MJ, Bartz D, Watson KL. Abortion during the Covid-19 Pandemic—Ensuring Access to an Essential Health Service. N Engl J Med. 2020;382(19):e47. doi: 10.1056/NEJMp2008006 32272002

[7] Romanis EC, Parsons JA. Legal and policy responses to the delivery of abortion care during COVID-19. Int J Gynaecol Obstet. 2020;151(3):479–486. doi: 10.1002/ijgo.13377 32931598 PMC9087790

[8] Endler M, Al‐Haidari T, Benedetto C, et al. How the coronavirus disease 2019 pandemic is impacting sexual and reproductive health and rights and response: Results from a global survey of providers, researchers, and policy‐makers. Acta Obstet Gynecol Scand. 2021;100(4):571–578. doi: 10.1111/aogs.14043 33179265 PMC8247356

[9] Tuppin P, Rudant J, Constantinou P, et al. Value of a national administrative database to guide public decisions: From the système national d’information interrégimes de l’Assurance Maladie (SNIIRAM) to the système national des données de santé (SNDS) in France. Revue d’Épidémiologie et de Santé Publique. 2017;65:S149–S167. doi: 10.1016/j.respe.2017.05.004 28756037

[10] Matulonga Diakiese B, Féron V. Induced abortion and COVID-19: What changed with the pandemic in 2020. Rev Epidemiol Sante Publique. 2022;70(6):277–285. doi: 10.1016/j.respe.2022.06.310 36123204 PMC9452417

[11] Vilain A, Rey S, Le Ray C, Quantin C, Zeitlin J, Fresson J. Impact of the COVID-19 pandemic on induced abortions in France in 2020. Am J Obstet Gynecol. 2022;226(5):739–741.e1. doi: 10.1016/j.ajog.2021.12.265 34999085 PMC8824736

[12] Vilain A, Fresson J, Rey S. Interruptions volontaires de grossesse: une légère baisse du taux de recours en 2020. Études et Résultats. 2021;(1207). https://drees.solidarites-sante.gouv.fr/publications/etudes-et-resultats/interruptions-volontaires-de-grossesse-une-legere-baisse-du-taux

[13] Breton D, Belliot N, Barbieri M, d’Albis H, Mazuy M, Dutreuilh C. Recent Demographic Trends in France: The Disruptive Impact of COVID-19 on French Population Dynamics: Fewer Births and Marriages, a Downturn in Migration, More Deaths…. Population. 2021;76(4):537–594.

[14] Breton D, Barbieri M, Belliot N, d’Albis H, Mazuy M. Recent demographic trends in France: situations and behaviour of minors. Population. 2020;75(4):467–526. doi: 10.3917/popu.2004.0467

[15] Bojovic N, Stanisljevic J, Giunti G. The impact of COVID-19 on abortion access: Insights from the European Union and the United Kingdom. Health Policy. 2021;125(7):841–858. doi: 10.1016/j.healthpol.2021.05.005 34052058 PMC8674116

[16] Parsons JA, Romanis EC. 2020 developments in the provision of early medical abortion by telemedicine in the UK. Health Policy. 2021;125(1):17–21. doi: 10.1016/j.healthpol.2020.11.006 33239186 PMC8847102

[17] Atay H, Perivier H, Gemzell-Danielsson K, et al. Why women choose at-home abortion via teleconsultation in France: drivers of telemedicine abortion during and beyond the COVID-19 pandemic. BMJ Sex Reprod Health. 2021;47(4):285–292. doi: 10.1136/bmjsrh-2021-201176 34321255

[18] Skuster P, Dhillon J, Li J. Easing of Regulatory Barriers to Telemedicine Abortion in Response to COVID-19. Front Glob Womens Health. 2021;2:705611. doi: 10.3389/fgwh.2021.705611 34901929 PMC8652224

[19] Harden J, Ancian J, Cameron S, Boydell N. Women’s experiences of self-administration of misoprostol at home as part of early medical abortion: a qualitative evaluation. BMJ Sex Reprod Health. 2021;47(2):144–149. doi: 10.1136/bmjsrh-2020-200661 32718985

[20] van Ooijen LT, Gemzell-Danielsson K, Waltz M, Gomperts R. A trans-national examination of the impact of the COVID-19 pandemic on abortion requests through a telemedicine service. BMJ Sex Reprod Health. 2022;48(3):179–184. doi: 10.1136/bmjsrh-2021-201159 34725053 PMC8561822

[21] Kapp N, Lohr PA. Modern methods to induce abortion: Safety, efficacy and choice. Best Practice & Research Clinical Obstetrics & Gynaecology. 2020;63:37–44. doi: 10.1016/j.bpobgyn.2019.11.008 32029379

[22] Cameron S. Recent advances in improving the effectiveness and reducing the complications of abortion. F1000Research. 2018;7:1881. doi: 10.12688/f1000research.15441.1 30631424 PMC6281004

