# Supplementary material for: Medication abortion during the COVID-19 pandemic in France: A research based on the French national health insurance database
Source: PLoS One. 2024 Feb 7;19(2):e0295336. doi: 10.1371/journal.pone.0295336 (PMC10849394; doi:10.1371/journal.pone.0295336)
Supplement: S1 Table — Notes: Coverage: abortions in 2019 and 2020 in the whole of France. Source: Authors’ calculations based on the French health insurance database (PMSI-MCO & CNAM). In 2020, the total number of abortions may include a small number of acts related to one single woman. The unit of analysis is based on all care procedures that were covered, rather than on individuals. (DOCX) [file pone.0295336.s001.docx]

**S1 Table. Annual abortions by setting and technique in 2019 and 2020.**

|  | 2019 | % | 2020 | % | Change  2019–2020 |
| --- | --- | --- | --- | --- | --- |
| Surgical abortions | 69,186 | 29 | 57,346 | 25 | -17% |
| Medical abortions in hospital settings | 102,086 | 44 | 100,440 | 45 | -2% |
| Medical abortions in non-hospital settings | 62,793 | 27 | 68,164 | 30 | +9% |
| Total | 234,065 | 100 | 225,950 | 100 | -3% |

*Notes:* Coverage: abortions in 2019 and 2020 in the whole of France.

Source: Authors’ calculations based on the French health insurance database (PMSI-MCO & CNAM).

In 2020, the total number of abortions may include a small number of acts related to one single woman. The unit of analysis is based on all care procedures that were covered, rather than on individuals.
