# Supplementary material for: Medication abortion during the COVID-19 pandemic in France: A research based on the French national health insurance database
Source: PLoS One. 2024 Feb 7;19(2):e0295336. doi: 10.1371/journal.pone.0295336 (PMC10849394; doi:10.1371/journal.pone.0295336)
Supplement: S2 Table — * Homogeneous groups of patients (Groupe homogène de malades, GHM) are a French administrative classification used to measure hospitals’ activity. They are the similar to the Diagnosis related groups (DRG) used in the United States. ** The Classification commune des actes médicaux (CCAM) is the French classification of medical procedures. (DOCX) [file pone.0295336.s002.docx]

**S2 Table. List of codes for abortions.**

| **French national health insurance database variables** | **Code** | **Description** |
| --- | --- | --- |
| ***Hospital setting*** | | |
| **Main diagnosis**  **(ICD-10)**  *DGN_PAL* | O0400 | Medical abortion [elective abortion in the legal framework]: incomplete, complicated by genital tract and pelvic infection |
|  | O0410 | Medical abortion [elective abortion in the legal framework]: incomplete, complicated by delayed or excessive haemorrhage |
|  | O0420 | Medical abortion [elective abortion in the legal framework]: incomplete, complicated by embolism |
|  | O0430 | Medical abortion [elective abortion in the legal framework]: incomplete, with other and unspecified complications |
|  | O0440 | Medical abortion [elective abortion in the legal framework]: incomplete, without complication |
|  | O0450 | Medical abortion [elective abortion in the legal framework]: complete or unspecified, complicated by genital tract and pelvic infection |
|  | O0460 | Medical abortion [elective abortion in the legal framework]: complete or unspecified, complicated by delayed or excessive haemorrhage |
|  | O0470 | Medical abortion [elective abortion in the legal framework]: complete or unspecified, complicated by embolism |
|  | O0480 | Medical abortion [elective abortion in the legal framework]: complete or unspecified, with other and unspecified complications |
|  | O0490 | Medical abortion [elective abortion in the legal framework]: complete or unspecified, without complication |
| **Homogeneous groups of patients** *  *GRG_GHM* | 14Z08Z | Elective abortion: less than three-days’ stay |
| **Classification of medical procedures** ** *CDC_ACT* | JNJP001 | Evacuation of a gravid uterus using medication during the 1st trimester of pregnancy |
|  | JNJD001 | Evacuation of a gravid uterus using medication, during the 2nd trimester of pregnancy and before the 22th week of gestation |
|  | JNJD002 | Evacuation of a gravid uterus by vacuum aspiration and/or curettage during the 1st trimester of pregnancy |
| ***Non-hospital setting*** | | |
| **Social security reimbursement** *PRS_NAT_REF* | 3329 | Flat fee for a medical abortion performed in a non-hospital setting |

* Homogeneous groups of patients (G*roupe homogène de malades, GHM*) are a French administrative classification used to measure hospitals’ activity. They are the similar to the Diagnosis related groups (DRG) used in the United States.

** The *Classification commune des actes médicaux* (*CCAM*) is the French classification of medical procedures.
